# Spinal Ependymoma Identified Following Spinal Anesthesia for Cesarean Delivery

**DOI:** 10.7759/cureus.12558

**Published:** 2021-01-07

**Authors:** Amber N Quintana, Richesh Guragain, Sophie Dean, Adam Moore, Linden Lee

**Affiliations:** 1 Anesthesiology, McGovern Medical School, University of Texas Health Science Center, Houston, USA

**Keywords:** ependymoma, epidural hematoma, neuraxial anesthesia, spinal anesthesia, spinal cord tumor

## Abstract

Neuraxial anesthesia is preferred over general anesthesia for cesarean delivery when appropriate because the latter is associated with a higher incidence of airway complications and an increased need for neonatal resuscitation. Common complications of neuraxial anesthesia include backache and headache, whereas feared but rare complications include paraplegia, intraspinal hemorrhage, cauda equina syndrome, nerve injury, and epidural hematoma. This case report presents a 40-year-old female with undiagnosed and asymptomatic ependymoma who presented with concerning neurological symptoms after receiving spinal anesthesia for elective cesarean delivery. Stat lumbar and thoracic spine magnetic resonance imaging (MRI) were performed on postoperative day 13 and were suggestive of a large hypervascular mass with areas of hemorrhage, concerning for ependymoma. An emergent laminectomy and mass resection were performed, resulting in an improvement in the patient’s neurological symptoms.

## Introduction

Neuraxial anesthesia is commonly performed for obstetric indications such as vaginal or cesarean delivery, tubal ligation, and cervical cerclage placement. Neuraxial anesthesia is generally safe, with common potential complications that include backache, post-dural puncture headache, and hypotension resulting in dizziness, nausea, and vomiting [1]. Rare complications include intraspinal hemorrhage, cauda equina syndrome, nerve injury, epidural abscess, and epidural hematoma, which may result in permanent nerve damage. The incidence of spinal epidural hematoma is 0-0.6 per 100,000 epidural catheterizations [2]. Bleeding complications within the central nervous system (CNS) can lead to permanent neurologic deficits if not recognized and treated promptly. Anesthesiologists should seek urgent evaluation in the setting of radicular back pain or bladder or bowel dysfunction that may indicate an expanding lesion [3]. Ependymomas are rare CNS tumors, constituting about 1.8% of all CNS tumors, with an overall incidence rate of 0.41/100,000 [4]. If symptomatic, patients may present with pain, sensory symptoms, weakness, bladder or bowel dysfunction, with symptoms lasting for an average of 8 months prior to diagnosis [5, 6]. Herein, we report a case of a 40-year-old female with a delayed presentation of an undiagnosed ependymoma who presented with neurological symptoms of headache, radicular back pain and new bladder dysfunction after receiving spinal anesthesia for cesarean delivery. In addition, we reviewed the cases in the literature where patients with asymptomatic ependymomas presented with clinical symptoms after neuraxial procedures. This article was previously presented as a poster at the 2020 American Society of Anesthesiologists Annual Meeting on October 3, 2020.

## Case presentation

A 40-year-old G6P5 female with a history of a torn left meniscus presented for a scheduled primary cesarean delivery and bilateral tubal ligation due to breech presentation. She had delivered vaginally five times before, each uneventfully with a labor epidural. Spinal anesthesia was performed at the L3-L4 level with a 25g Whitacre needle (BD, Franklin Lakes, NJ, USA) in one attempt without difficulty. 12mg of 0.75% hyperbaric bupivacaine, 15mcg of fentanyl, and 100mcg of morphine were administered intrathecally. The cesarean delivery was uneventful and the patient was discharged home on postoperative day three. The patient presented on postoperative day six with new-onset low back pain with radiation to the lumbar paraspinals and posterior right thigh. The patient reported a history of spontaneously resolving sciatica symptoms in the postpartum period after receiving epidurals in prior pregnancies. The patient denied incontinence of urine or bowel and her neurologic exam showed no new weakness. She reported mild weakness in her left lower extremity that had been present prior to neuraxial anesthesia and was thought to be from her torn left meniscus. The pain was alleviated by hydrocodone and ibuprofen. The clinical picture was not consistent with a neuraxial abscess or hematoma and our differential diagnosis at the time included musculoskeletal pain and discogenic impairment of her lumbar spine, given her sciatica after prior deliveries. Given the benign neurological history and physical exam, a space-occupying lesion was low in the differential diagnosis. The patient was discharged home with a recommended trial of gabapentin for pain management.

**Figure 1:**
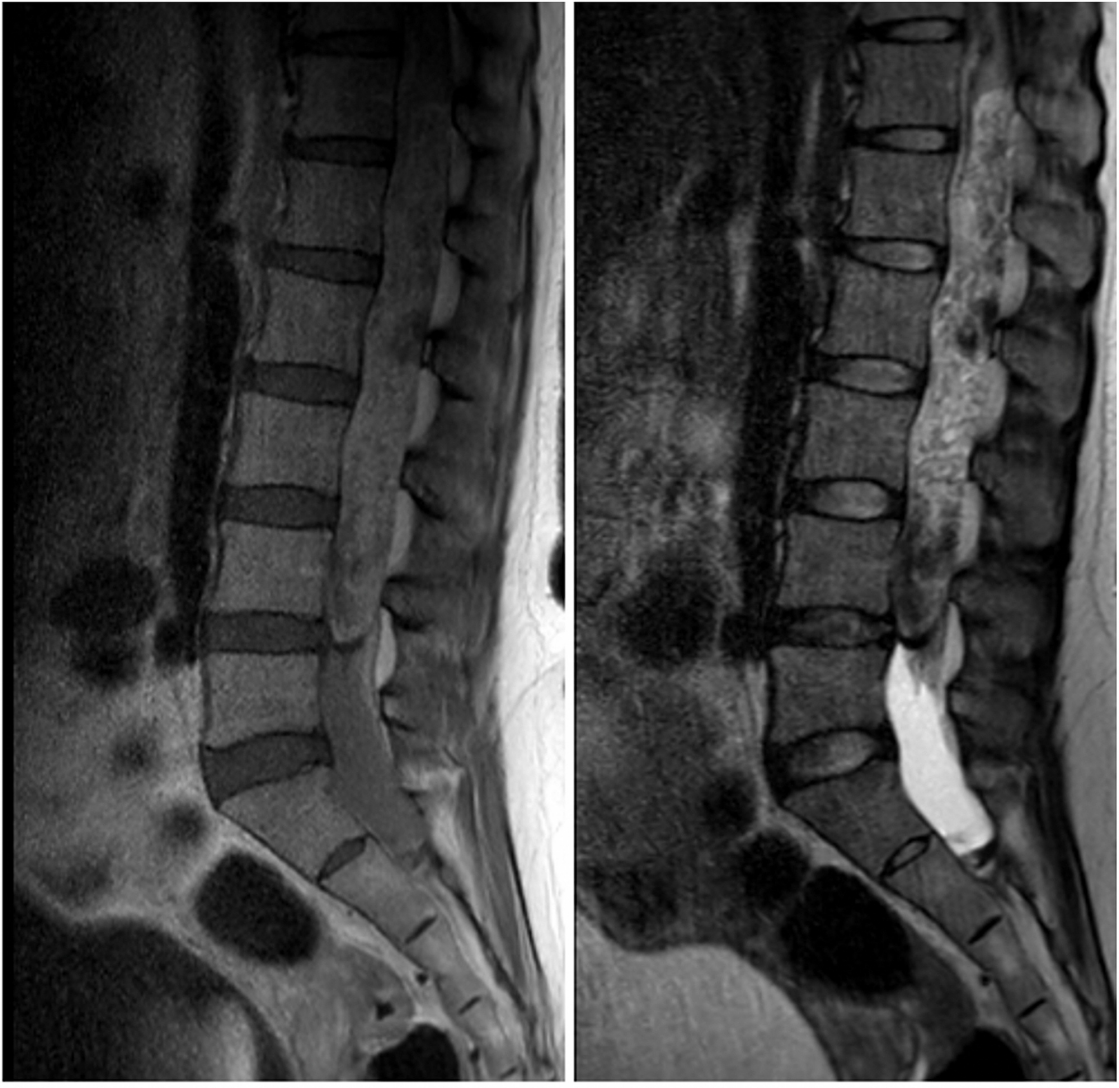
Initial lumbar magnetic resonance image (MRI) scan. T1-weighted MRI (left). T2-weighted MRI (right). There are extensive T2 signal hypointensities within the mass, representing small vascular structures and blood products.

The patient subsequently returned on postoperative day 13 for new-onset urinary retention and difficulty ambulating. Her neurologic examination was remarkable for numbness in the bilateral lower extremities, 3/5 strength on hip flexion, and 4/5 strength on plantar and dorsiflexion bilaterally. Stat lumbar and thoracic spine magnetic resonance imaging (MRI) exams were obtained and revealed a large hypervascular mass with areas of hemorrhage occupying and expanding the spinal canal from T11 through L4-L5 junction with a small amount of subarachnoid hemorrhage in the sacral canal (Figure 1).

**Figure 2:**
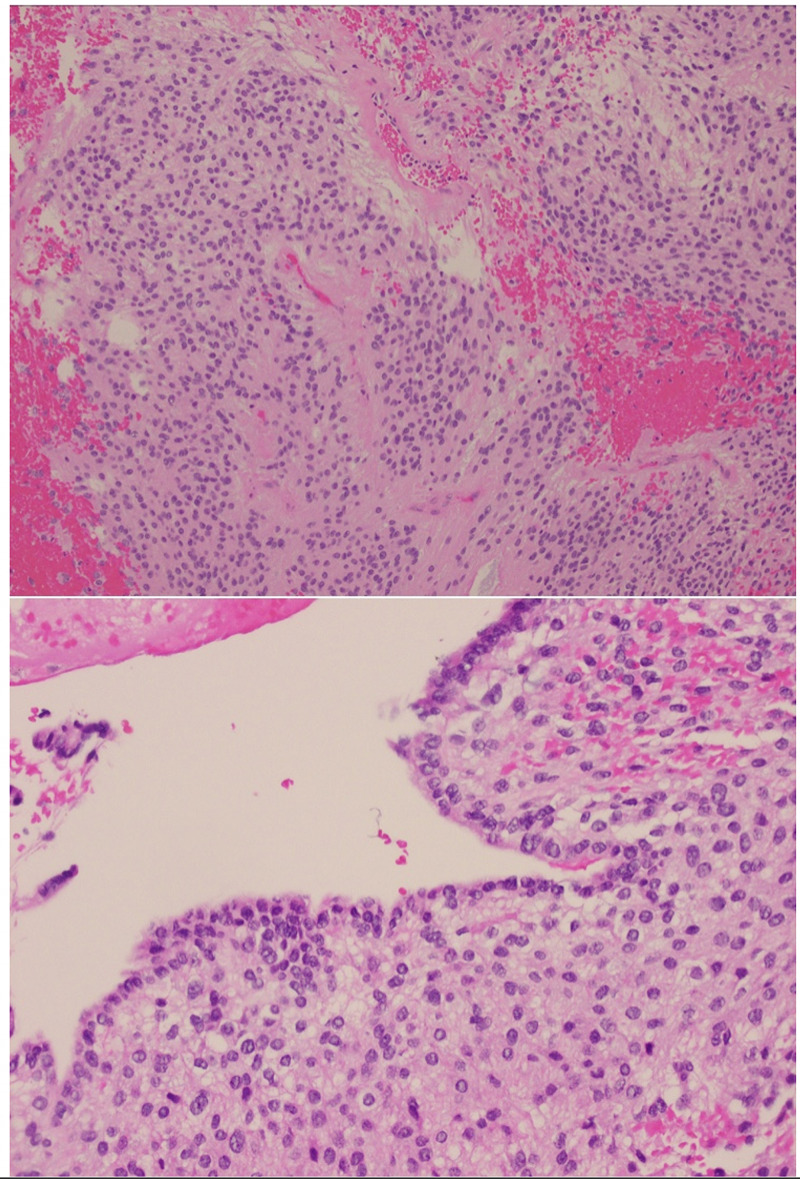
World Health Organization (WHO) grade II ependymoma. Tumor cells form perivascular pseudorosettes (top) and ependymal canals (bottom).

Neurosurgery performed an emergent T11-L5 laminectomy for mass resection. Surgical pathology confirmed the diagnosis of myxopapillary ependymoma (Figure 2).

She was discharged to inpatient rehabilitation on postoperative day four with residual but improved lower extremity weakness and neurogenic bowel and bladder. While in inpatient rehabilitation, the patient’s neurogenic bowel and neurogenic bladder improved, with urology recommendation to continue intermittent straight catheterization daily to twice daily. She continued to have lower extremity weakness, although this improved during her rehabilitation admission. She was subsequently discharged home with outpatient physical therapy and occupational therapy.

## Discussion

Spinal cord ependymomas are rare and generally slow-growing tumors with complete surgical resection being the first-line treatment. Patients with spinal cord ependymomas usually present in their 30s-40s with nonspecific symptoms that may progress over years prior to diagnosis. However, intratumoral hemorrhage can provoke acute deterioration. Neuraxial anesthesia may be a factor in unmasking symptoms due to local trauma and intratumoral hemorrhage. Depending on the location, ependymomas may cause upper or lower extremity symptoms if present in the cervical area, or incontinence, radicular back and leg pain, and asymmetric weakness if present in the lumbar area [7].

Five prior cases were described in the literature of women who underwent neuraxial anesthesia for delivery, followed by neurologic symptoms and subsequent discovery of spinal ependymoma (Table 1). Campbell et al. described a case of hemorrhage from an occult spinal ependymoma after an uncomplicated epidural placement [10]. Jaeger et al. described a case of a lumbar ependymoma that was discovered after neurological symptoms arose following an unsuccessful spinal attempt, with an MRI revealing intraspinal bleeding. Jaeger et al. postulated that the increased intradural pressure may have aggravated the onset of symptoms [3]. Similarly, Lee et al. described a case of acute paraplegia after an uncomplicated spinal that led to the discovery of a spinal ependymoma. Lee et al. discussed that the internal bleeding may be attributed to either the acute decline in abdominal pressure due to the patient’s cesarean delivery, or due to the rapid change of intradural pressure [6]. As seen in Table 1, the previous cases presented young women in their 20s with the onset of acute neurological symptoms ranging from immediately after delivery to one week after neuraxial anesthesia.

**Table 1:** Reported cases of ependymomas in postpartum patients presenting with acute neurological symptoms

Author (Year)	Patient	Surgery	Anesthesia	Tumor	Onset of Symptoms	Treatment	Neurologic Deficits
Roscoe et al.(1984) [8]	24, female	Caesarean section	Epidural	Ependymoma	4 days	L1-L4 laminectomy	Residual motor weakness
Martin et al.(1992) [9]	31, female	Caesarean section	Epidural	Ependymoma	Immediately postoperatively	L4-L5 laminectomy	Full recovery
Jaeger et al.(2002) [3]	24, female	Caesarean section	Unsuccessful spinal attempt, general anesthesia	Ependymoma with massive intratumoral hemorrhage	12 hours	T12-L2 laminotomy	Improving motor function, absent bladder function
Campbell et al. (2008) [10]	27, female	Caesarean section	Epidural	Ependymoma with intratumoral hemorrhage	3 days	T12-L2 laminectomy	Full recovery
Lee et al.(2016) [6]	24, female	Caesarean section	Spinal	Ependymoma with hemorrhage	Immediately after delivery	Left C6-T2 unilateral hemilaminectomies	Residual motor deficit and cuada equina syndrome
Present report (2020)	40, female	Caesarean section	Spinal	Ependymoma with hemorrhage	6 days	T11-L5 laminectomy	Residual motor weakness

## Conclusions

Our patient likely had this large preexisting asymptomatic ependymoma that was discovered when spinal anesthesia caused localized trauma, hematoma, and progressive neurological symptoms. Localized trauma and hemorrhage following neuraxial anesthesia may be a common factor in acute deterioration within a week in these otherwise healthy young patients as tumors may be more vascular and likely to bleed than the usual CNS contents. As undiagnosed asymptomatic CNS tumors are rare and it is not feasible to screen all parturients prior to neuraxial anesthesia, this case and the previously reported cases highlight the importance of prompt evaluation of worsening neurologic symptoms after neuraxial anesthesia. Following this incident, to improve the quality of care and patient education, we have revised our discharge instructions to include a special section on concerning symptoms following neuraxial anesthesia that patients should return to be evaluated for. We hope that this will allow patients to receive timely evaluation and treatment to potentially avoid permanent neurological sequelae. 
